# Application of thyroglobulin and anti-thyroglobulin antibody combined with emission computed tomography in the adjuvant diagnosis of differentiated thyroid carcinoma

**DOI:** 10.1080/07853890.2023.2232372

**Published:** 2023-07-12

**Authors:** Nan Jiang, Benzheng Jiao, Laney Zhang, Jialong Li, Yungeng Li, Chenghe Lin

**Affiliations:** a Department of Nuclear Medicine, The First Hospital of Jilin University, Changchun, PR China; b Yale School of Public Health, New Haven, CT, USA; c Division of Biosciences, University College London, London, UK

**Keywords:** Differentiated thyroid carcinoma, thyroglobulin, anti-thyroglobulin antibody, radionuclide imaging, diagnostic value

## Abstract

**Purpose:**

Thyroid nodules are a kind of common endocrine system disease, with approximately 5% of them developing into malignant lesions, the most common of which belong to differentiated thyroid carcinoma (DTC). Accurate differential diagnosis using reliable methods and targeted treatment of benign and malignant thyroid nodules are of great significance to improve patient outcomes. This study mainly investigates the diagnostic value of thyroglobulin (Tg) and anti-thyroglobulin antibody (anti-TgAb) combined with emission computed tomography (ECT) in the adjuvant diagnosis DTC.

**Methods:**

All the data of 387 histopathologically diagnosed DTC patients (observation group) and 151 patients with nodular goiter (control group) admitted between June 2019 and June 2021 were collected and retrospectively analyzed. Serum Tg and anti-TgAb levels were detected in all subjects. In addition, all patients in the observation group underwent thyroid ECT, and the results were compared with the pathological findings. The receiver operating characteristic (ROC) curve was drawn to analyze the diagnostic performance of Tg, TgAb and thyroid ECT, either alone or in combination, in patients with thyroid cancer (TC).

**Results:**

The consistency test showed that Tg (Kappa-value = 0.370) and anti-TgAb (Kappa-value = 0.393) had generally consistent efficiency with pathological findings in the diagnosis of DTC; ECT (Kappa-value = 0.625) and the combined diagnosis of the three (Kappa-value = 0.757) showed higher consistency than the pathological diagnosis, of which the combined diagnosis contributed to an even higher consistency. The combined diagnosis of Tg, anti-TgAb, and thyroid ECT outperformed either of these alone in DTC diagnosis, with a sensitivity of 91.5%, a specificity of 86.1%, and an accuracy of 90%.

**Conclusions:**

The combination of Tg. anti-TgAb, and RNI can effectively improve the diagnostic accuracy of DTC and reduce the missed diagnosis rate, which has important reference significance for clinical diagnosis and treatment of TC.

## Introduction

Thyroid cancer (TC) is a common type of endocrine malignancies, accounting for approximately 95% of all endocrine tumors and 2.5% of all malignant tumors [1,2,3]. TC is now considered a major public health issue worldwide due to its rapidly rising incidence [4,5], with papillary (70–75%) and follicular (15–20%) [6] TC being the most common pathological types. Papillary thyroid cancer (PTC) arises from thyroid follicular cells with the papillary structures encompassing tumor epithelium overlying a true fibrous vascular stalk, termed differentiated thyroid carcinoma (DTC) [7]. PTC is rarely present as a homogeneous tumor and has various histopathological variants [8,9]. Intertumor heterogeneity refers to genetic variations that occur between individuals with the same tumor type. TC heterogeneity is not limited to phenotypic diversity but also manifests itself as genetic variation [10]. Genome analysis of TC revealed complex mutations and enormous inter-and intratumoral heterogeneity [11].

At present, the pathogenesis of TC has not been thoroughly clarified clinically. After the onset, neck mass, decreased blood calcium, diarrhea, hoarseness, dyspnea and dysphagia are the main clinical presentations, affecting the healthy life of patients [12]. Although DTC carries a favorable prognosis, its clinical and biological behavior is relatively slow, resulting in frequent neck lymph node metastasis at diagnosis or during postoperative follow-up [13]. Therefore, preoperative diagnosis and regular postoperative follow-up are particularly important. Pathological examination, a commonly used diagnostic method for TC, is considered as the "gold standard" for the disease. However, it is risky and traumatic, leading to poor patient tolerance for diagnosis [14]. Among the non-invasive diagnostic modalities, ultrasound [15] is most extensively used for early screening of thyroid diseases. However, due to the occlusion of neck muscle tissue and the limited sensitivity of ultrasound itself, its value in early warning or diagnosis of DTC recurrence and metastasis is not high. In recent years, the role of emission computed tomography (ECT) technology in the diagnosis of TC has gradually attracted widespread clinical attention. It is an examination method using radionuclides that, after computer processing, can image the difference in radioactivity concentration between the lesion and normal tissue, providing valuable imaging information for disease diagnosis [16,17].

In addition, the combined detection of serum thyroglobulin (Tg) and anti-thyroglobulin antibody (anti-TgAb) has been indicated to be helpful for postoperative follow-up of TC patients and prediction of recurrence risk [18]. Tg is a thyroid-specific protein synthesized by thyroid follicular epithelial cells and stored in the thyroid follicular lumen, with extremely low content in human circulating blood under physiological conditions [19]. While the level of serum TgAb has been indicated to be closely related to TC [20]. Therefore, the purpose of this paper is to explore the diagnostic value of Tg and anti-TgAb alone and in combination with radionuclide imaging (RNI) in DTC.

## Data and methods

### Screening criteria and research subjects

This is a retrospective analysis. We selected patients according to the following criteria: (1) meeting the diagnostic criteria for DTC and benign thyroid diseases, with pathologically confirmed diagnosis; (2) available serum Tg and antiTgAb test results; (3) available ECT results in all TC patients; (4) complete clinicopathological and follow-up data.

In contrast, cases were excluded if they met any of the following criteria: (1) other primary malignant tumor diseases; (2) acute/chronic infection or severe heart, liver, renal insufficiency and other diseases; (3) mental disorders, cognitive impairment or organic diseases; (4) incomplete clinicopathological data.

**Figure 1.**
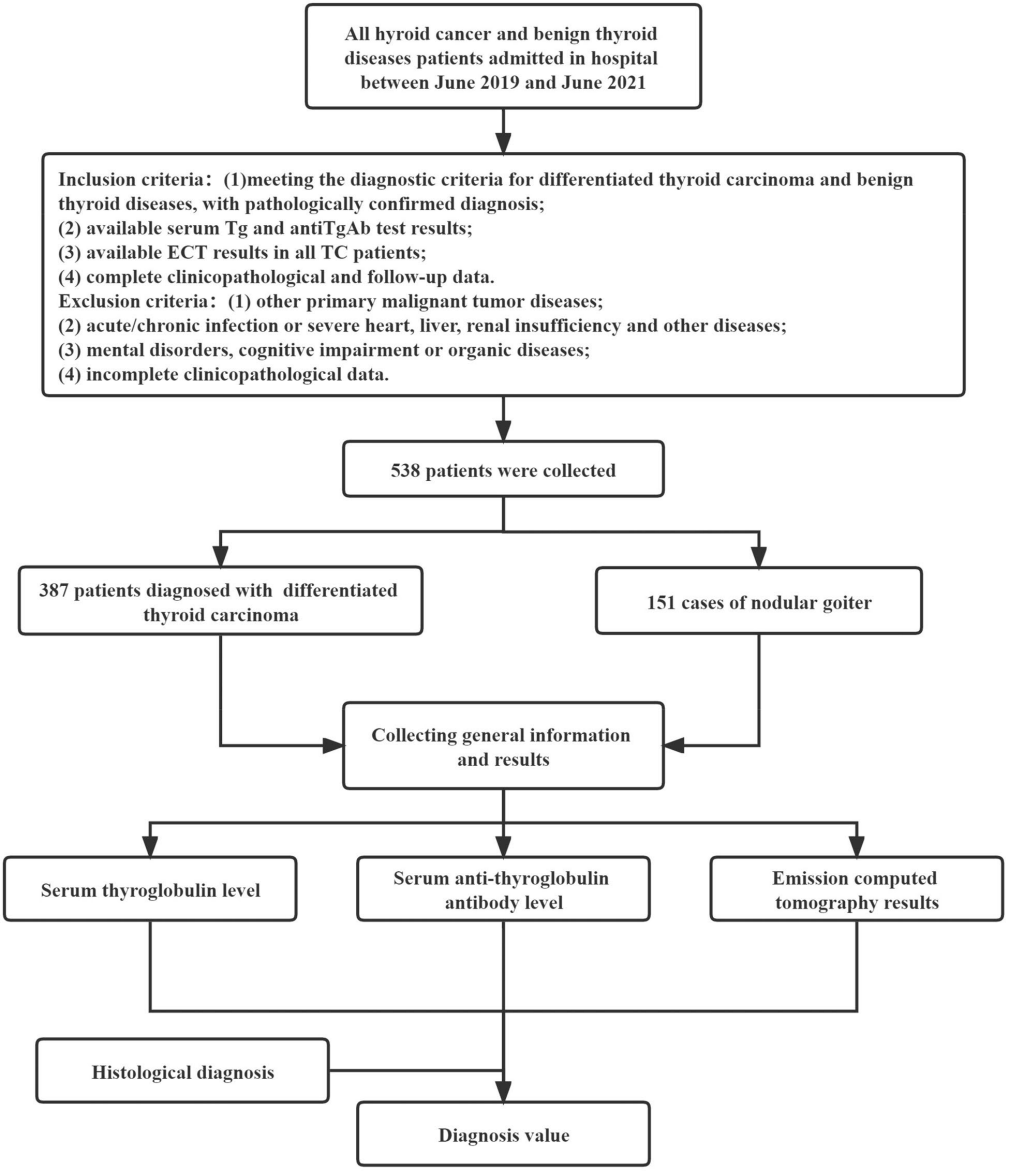
Schematic flow-chart of the study. Tg: thyroglobulin; anti-TgAb: anti-thyroglobulin antibody; ECT: emission computed tomography.

Based on the above criteria, we selected 387 cases from all DTC patients admitted to The First Hospital of Jilin University between June 2019 and June 2021 as the observation group. Additionally, 151 concurrent patients with nodular goiter were selected as the control group. 93 patients underwent fine-needle aspiration biopsy to confirm the diagnosis. The study was approved by the ethics committee of The First Hospital of Jilin University (202106). This is a retrospective study, and because the analysis uses anonymous clinical data approved by the Ethics Committee of The First Hospital of Jilin University, subjects or guardians do not need to give informed consent to the study. The schematic flow-chart of the study is shown in Figure 1.

### Collection and detection of serum specimens

From all participants, fasting venous blood was sampled in the early morning and centrifuged (4500 r/min, 10 min) after 30 min of standing. After serum separation, the Tg level was determined by automatic chemiluminescence immunoassay analyzer (Roche, Germany). The level of anti-TgAb in each group was determined with the same automatic chemiluminescence immunoassay analyzer and related supporting reagents. The normal reference range of serum Tg was 1.4–78.0 ng/mL, and a detection level > 78.00 ng/mL was considered positive (Y. Hu, Yin, Liu, Xiu, & Shi). Serum TgAb over 115 IU/mL was defined as positive [21].

### Thyroid ECT

Thyroid ECT was performed in both groups. Before the examination, the relevant knowledge of ECT examination was explained to patients and their family members, and the matters needing attention during the examination were informed to improve patient cooperation. A Single-Head ECT scanner produced by Siemens, Germany, and equipped with an ultra-high-energy collimator, was used, with parameters set as follows: matrix: 256 × 256, the acquisition count: 5 × 10^5^, magnification: × 3.2. The participant was lying in the supine position with the neck in hyperextension, and radioisotope Technetium-99m (^99m^Tc) was injected through the elbow vein. Twenty minutes later, the scanning examination was initiated, and the anterior and posterior thyroid imaging examinations were performed to observe the distribution of isotope radioactivity in the thyroid, mass and surrounding tissues. The functional status of thyroid lesions was evaluated as either cold, cool, warm or hot: cold nodules were thyroid lesions without radioactive distribution; cool nodules referred to thyroid lesions with a little radioactive distribution; warm nodules were defined as thyroid lesions with the same radioactivity distribution as normal thyroid tissue; hot nodules referred to thyroid lesions with a higher distribution than normal thyroid tissue.

Judgment criteria: cold and cool thyroid nodules during thyroid ECT examination, with irregular and fuzzy edges were suspected of TC, while warm and hot nodules were benign thyroid lesions. The inspection results are compared with the gold standard. In the diagnosis of DTC with Tg and anti-TgAb combined ECT, the diagnosis was positive if two or more test results were positive, and negative if all the three tests were negative.

### Statistical analysis

Statistical analysis of the data was performed with SPSS 20.0. n(%) and Mean ± SD were used to indicate count and measurement data, respectively, with their inter-group comparison methods being the chi-square test and the independent samples t test, respectively. The Kappa-test was performed for consistency analysis, with a Kappa-value of 0.21–0.40, 0.41–0.60, and 0.61–0.80 indicating general, medium, and high consistency, respectively; a greater Kappa-value indicates better consistency. The receiver operating characteristic (ROC) curve was plotted and the area under the curve (AUC) was calculated. *p* < 0.05 is indicative of statistical significance.

## Results

### General information of the selected patients

As can be found in Table 1, the two cases series showed no marked difference in general data (gender, age, etc.); however, the levels of Tg and TgAb of the two groups were significantly different (*p* < 0.05).

**Table 1.** Baseline data.

Indicators	Observation group (*n* = 387)	Control group (*n* = 151)	χ^2^/t	*p* Value
Gender			1.299	0.254
Male	189 (48.8)	82 (54.3)		
Female	198 (51.2)	69 (45.7)		
Age (years old)	42.7 ± 6.8	43.2 ± 7.0	0.760	0.448
Nodule diameter (cm)	2.97 ± 0.39	3.02 ± 0.36	1.365	0.173
Tumor types				
Papillary thyroid carcinoma	299 (77.3)	–		
Follicular thyroid carcinoma	88 (22.7)	–		
Tg (ng/mL)	161.96 ± 56.21	67.75 ± 23.47	19.920	< 0.0001
TgAb (IU/mL)	191.37 ± 50.60	105.10 ± 36.12	19.139	< 0.0001

Tg: thyroglobulin; anti-TgAb: anti-thyroglobulin antibody.

### Consistency of Tg, anti-TgAb and RNI diagnosis with pathological diagnosis

According to consistency test results (Tables 2–5), the diagnosis of DTC made by Tg and anti-TgAb showed general consistency with the pathological findings, with a Kappa-value of 0.370 and 0.393, respectively (*p* < 0.05); ECT diagnosis of DTC were in high consistency with the pathological diagnosis, with a Kappa-value of 0.625 (*p* < 0.05); the combination of the three exhibited the highest consistency with the pathological diagnosis, with a Kappa-value of 0.757.

**Table 2.** Comparison of thyroglobulin diagnosis and pathological results.

Thyroglobulin	Pathological results		Total	Kappa-value	Consistency degree
	Malignant tumor	Benign nodule			
Positive (> 78.00 ng/mL)	309 (57.4)	63 (11.7)	372	0.370	General
Negative (≤ 78.00 ng/mL)	78 (14.5)	88 (16.4)	166		
Total	387	151	538		

**Table 3.** Comparison of anti-thyroglobulin antibody diagnosis and pathological results.

Anti-thyroglobulin antibody	Pathological results		Total	Kappa-value	Consistency degree
	Malignant tumor	Benign nodule			
Positive (> 115 IU/mL)	323 (60.0)	67 (12.5)	390 (72.5)	0.393	General
Negative (≤ 115 IU/mL)	64 (11.9)	84 (15.6)	148 (27.5)		
Total	387 (71.9)	151 (28.1)	538 (100.0)		

**Table 4.** Comparison of ECT diagnosis and pathological results.

ECT	Pathological results		Total	Kappa-value	Consistency degree
	Malignant tumor	Benign nodule			
Malignant tumor	341 (63.4)	35 (6.4)	376 (69.8)	0.625	Relatively high
Benign nodule	46 (8.6)	116 (21.6)	162 (30.2)		
Total	387 (72.0)	151 (28.0)	538 (100.0)		

ECT: emission computed tomography.

**Table 5.** Comparison of thyroglobulin and anti-thyroglobulin antibody combined with ECT diagnosis and pathological results.

Combined detection	Pathological results		Total	Kappa-value	Consistency degree
	Malignant tumor	Benign nodule			
Malignant tumor	354 (65.8)	21 (3.9)	375 (69.7)	0.757	High
Benign nodule	33 (6.1)	130 (24.2)	163 (30.3)		
Total	387 (71.9)	151 (28.1)	538 (100.0)		

ECT: emission computed tomography.

### ROC curve analysis of Tg and anti-TgAb combined with ECT diagnosis

**Figure 2.**
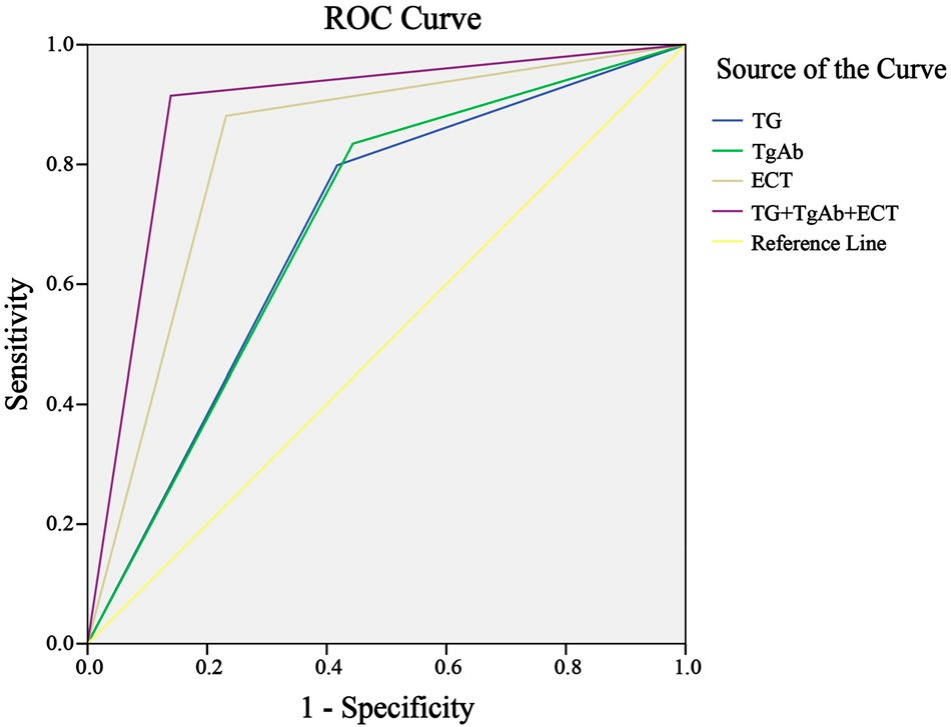
ROC Curves of different diagnostic methods. Tg: thyroglobulin; anti-TgAb: anti-thyroglobulin antibody; ECT: emission computed tomography.

According to ROC curve analysis, the combined detection of Tg, anti-TgAb and ECT had higher diagnostic value for DTC than either of them alone (*p* < 0.05), as shown in Figure 2 and Table 6.

**Table 6.** ROC curve analysis.

Diagnostic method	AUC	S.E	Sig	95%CI
Tg	0.691	0.027	0.000	0.638–0.743
TgAb	0.695	0.027	0.000	0.643–0.748
ECT	0.825	0.022	0.000	0.781–0.868
Combined detection	0.888	0.018	0.000	0.852–0.924

Tg: thyroglobulin; anti-TgAb: anti-thyroglobulin antibody; ECT: emission computed tomography.

### Diagnostic efficacy of Tg and anti-TgAb combined with ECT

As shown in Table 7, the sensitivity, specificity, and accuracy of Tg in diagnosing DTC were 79.8, 58.3, and 73.8, respectively, compared to 83.5, 55.6, and 75.7 for the anti-TgAb diagnosis, and 88.1, 75.9, and 83.8 for the ECT diagnosis; while the sensitivity, specificity, and accuracy of their combined diagnosis were 91.5, 86.1, and 90.0, respectively.

**Table 7.** Diagnostic efficacy of Tg, TgAb and ECT (alone or in combination) in patients with differentiated thyroid carcinoma.

Diagnostic method	Sensitivity (%)	Specificity (%)	Accuracy (%)
Tg	79.8	58.3	73.8
TgAb	83.5	55.6	75.7
ECT	88.1	75.9	83.8
Combined diagnosis	91.5	86.1	90.0

Tg: thyroglobulin; anti-TgAb: anti-thyroglobulin antibody; ECT: emission computed tomography.

## Discussion

Benign thyroid nodules and DTC share clinical symptoms and signs [22], and how to make an accurate differential diagnosis between them has become a clinical research hotspot. Imaging examinations are often used in clinical diagnosis of thyroid diseases, but the effect is mediocre. According to the related literature, serum Tg can be used as an effective index for differential diagnosis of DTC [23]. TgAb, a kind of G immunoglobulin, is a routine marker of thyroid autoimmunity. TgAb is more common in DTC and can interfere with Tg measurements used to monitor DTC recurrence or persistence [24].

By comparing the diagnostic value of serum Tg and TgAb for DTC, we found that the diagnosis made by the two had general consistency with the pathological findings. Serum Tg, which is specifically produced and secreted by benign or differentiated malignant thyroid cells, is considered as a highly sensitive and specific tumor marker after surgical resection of benign and malignant thyroid tissue and ^131^I ablation in DTC patients [7]. The Tg level is generally low in the general population. But in patients with TC, damage to thyroid tissue structure and physiological structure can easily induce inflammation and the apoptosis of thyroid tissue, leading to the activation of thyroid epithelial cells, thereby releasing more Tg. Thus, Tg is highly expressed in TC patients [25, 26]. While the presence of TgAb may affect the Tg level, reducing or increasing serum Tg levels [27]. After binding to Tg, TgAb can activate NK cells through the interaction of Fc receptors and bound antibodies, and attack the target cells, leading to the destruction of thyroid cells and affecting thyroid hormone synthesis [28]. Scholars [29] have also proposed that a significant reduction in TgAb level represents a good prognostic signal for DTC patients. Importantly, some studies show that there is a close relationship between the volume of thyroid tissue and the Tg level, pointing out that the smaller the thyroid tissue volume, the lower the stimulated Tg level [30].

Considering that Tg levels are affected by TgAb, some scholars have proposed to reverse the influence of TgAb using the Tg recovery test. The results, however, are not satisfactory due to the pathophysiological changes of patients and experimental techniques. The hook effect, a common feature of most immunoassays, occurs when the antigen level is high, which results in a solid support for the binding capacity of the antibody, leading to false negative results [31]. Therefore, in 2006, the National Academy of Clinical Biochemistry (NACB) Guidelines proposed to abandon the recovery test and directly test TgAb instead [32]. At this stage, how to eliminate the interference of TgAb on Tg level detection remains a conundrum to decipher. The thyroid gland is known to have the function of absorbing iodine and concentrating iodine. Radioactive iodine, which is mostly distributed in the thyroid gland after entering the human body, can not only display the thyroid gland morphology, but also measure the iodine absorption rate of the thyroid gland. However, studies have found that some TCs have poor ^131^I uptake function [33]. Therefore, based on the principle of radionuclides, ^131^I with poor uptake should be avoided so as not to affect the diagnostic performance. Through cubital vein injection of radioisotope ^99m^Tc, the ECT technique enables the formation of radioactive concentration differences between the lesion and the surrounding normal tissue, thereby providing imaging evidence for the diagnosis and differentiation of benign and malignant thyroid lesions. It is shown that due to the difference in radionuclide capacity between thyroid nodules and surrounding normal thyroid tissues, TCs have relatively low uptake capacity and are mostly manifested as cold and cool nodules [34]. In this study, we found that ECT alone was in good consistency with the pathological results in the diagnosis of DTC, with diagnostic sensitivity, specificity, and accuracy of 88.1%, 75.9%, and 83.8%, respectively, indicating that ECT can provide a reference for clinical diagnosis of DTC. Nonetheless, many studies have confirmed [35,36] that cold nodules with defective radioisotope distribution are usually malignant tumors, though not in all cases. Besides, ECT is unable to distinguish the cystic and solid nature of benign and malignant lesions, with some certain limitations in the diagnosis of TC.

Finally, we analyzed the diagnostic efficacy of Tg and anti-TgAb in combination with ECT, and found that the diagnostic results were highly consistent with the pathological results, with ROC curve analysis indicating its sensitivity, specificity, and accuracy being 91.5%, 86.1%, and 90.0%, respectively. This is because their combined diagnosis can make up for the deficiency of their single use, which is conducive to reducing missed diagnoses and improving the clinical diagnosis performance.

## Conclusion

The combined application of Tg, anti-TgAb and RNI can improve the diagnostic accuracy in TC and thus provide a basis for the formulation and implementation of disease treatment plans, which has certain clinical value for the diagnosis of TC.

## Data Availability

The labeled dataset used to support the findings of this study are available from the corresponding author upon request.
